# Employee Satisfaction With Working Space and Its Association With Well-Being—A Cross-Sectional Study in a Multi-Space Office

**DOI:** 10.3389/fpubh.2019.00358

**Published:** 2019-11-26

**Authors:** Sirpa Lusa, Sari Mirjami Käpykangas, Heli Ansio, Pia Houni, Jukka Uitti

**Affiliations:** ^1^Quantified Employee Program, Center of Expertise for Work Ability and Working Careers, Finnish Institute of Occupational Health, Tampere, Finland; ^2^Center of Expertise for Digital Transformation of Work, Finnish Institute of Occupational Health, Tampere, Finland; ^3^Finnish Institute of Occupational Health, Centre of Expertise for SMEs and Growth, Helsinki, Finland; ^4^Center of Expertise for Transforming Occupational Health Services, Finnish Institute of Occupational Health, Tampere, Finland; ^5^Faculty of Medicine and Health Technology, Tampere University, Tampere, Finland

**Keywords:** multi-space office, workspace satisfaction, well-being, communality, social capital

## Abstract

**Introduction:** Different kinds of shared and activity-based offices are common today and employees' experiences, perceived health, well-being, satisfaction, and productivity have been studied in different types and sizes of environments.

**Objectives:** In this study we aimed to determine employee satisfaction with a multi-space office. We also aimed to find associations between satisfaction with working space and both individual and social well-being.

**Methods:** A total of 91 employees from a multi-space office shared by six municipality-owned companies answered a self-administered questionnaire. Frequencies, percentages, averages, and minimum and maximum values are used to describe the results. We used cross-tabulation and Pearson's Chi-Square test to study the associations and linear regression analysis to create a model describing the variability of workspace satisfaction.

**Results:** The employees were most satisfied with the workspace furniture (82% of respondents) and most dissatisfied with workspace acoustics (44%). Workspace satisfaction was associated with self-satisfaction, good self-perceived future work ability, and good recovery. As regards social factors, workspace satisfaction was associated with good atmosphere among colleagues and good social capital. Satisfaction with acoustics was also associated with good self-perceived future work ability, recovery, and good social capital. Social capital best explained the general variation in workplace satisfaction.

**Conclusions:** Many individual- and social-related well-being outcomes, and especially social capital, were associated with workspace satisfaction. To maintain workplace satisfaction and well-being, attention should be paid to the design, functionality, and management of the used workspaces.

## Introduction

Different kinds of activity-based working environments are common in work life today (1). Among other things, employees' experiences, perceived health, well-being, job satisfaction, and productivity in environments of different type, and size have been studied previously (2–7). Studies have focused on interaction, communality, and comfort. It seems that employees are more satisfied working in single-rooms and next to multi-space offices. Employees in medium or large open space offices are the most dissatisfied. Those working in large open-space offices also have more sick leaves than those working in smaller offices (3).

According to several studies, the positive aspects of multi-space offices are their technical aspects, such as their aesthetic overall appearance, as well as effective information transfer, interaction, and communality. Some problems are related to technical solutions such as inadequate working or storage space, problems with mobile technology, or lack of privacy. Often, problems are also reported as concentration difficulties, interruptions, and irrelevant speech noise (2, 4–6, 8). Engelen et al. (6) stated in their extensive review that working in a multi-space office allows for better management of one's own work, but that the physical spaces are not properly designed for different purposes. These offices also require work community's psychosocial and leadership support.

Bodin Danielsson et al. (9) report that multi-space offices have the highest number of conflicts and disturbing noise. Researchers have emphasized the importance of taking acoustic design and social relationships into account when designing multi-space offices. Irrelevant speech noise (ISN) has shown to be the most disturbing source of noise in open-plan offices (10, 11), especially in offices over five employees (8). A study by De Been et al. (12) found that the positive aspects of working in multi-space offices were, for example, the opportunities to meet colleagues and have discussions, but also conversely, that in open-plan offices, discussions decreased, and social relationships weakened. Bernstein and Turban (13) also came to the same conclusion in their research, as they found that the transition to an open workspace significantly reduced face-to-face interaction and increased e-communications, contrary to expectations.

Lindberg et al. (14) found that those working in a common space, were more active and experienced lower perceived stress during the working day and lower measured physiological stress outside the office than those working in their own rooms or working in separated cubicles. It seems that certain types of working space solutions are reflected in workers' behavior; in this case, physical activity, and that they also lead to physiological changes during free time and increase well-being. Employees are physically active when they have to move to different workspaces to do different work tasks. However, the transition from one space to another must be smooth (15). Gerdenitch et al. (16) studied employees perceived need–supply fit in an activity-based office environment. According to them, both interaction across teams and workspace satisfaction increased after redesigning a cellular office as an activity-based office, especially for employees who reported high perceived need-supply fit (perception about the working environment supporting their needs).

It has been established that cleanliness, natural lightning, orientation, and regulation of lighting of the workspace have significant impact on the satisfaction of employees with any kind of the workspace (17). Some indoor environmental quality (IEQ) factors have differential impact on overall workspace satisfaction depending on whether they are considered satisfactory by space users or not. Adapting Kano's model on customer satisfaction, Kim and de Dear (18) label some of IEQ “Basic Factors”. They often go unnoticed when they perform well, but when they do not, they cause dissatisfaction. Kim and de Dear (18) identified temperature, noise level, amount of space, visual privacy, adjustability of furniture, colors, and textures, and cleanliness as such basic factors. The factors were ranked according to their strengths of impact on overall satisfaction. Amount of space is the factor that clearly makes the greatest impact on overall satisfaction both when it meets expectations and when it does not. The next most important factors are noise level, ease of interaction, and visual privacy, when their perceived performance is satisfactory. However, when the factors fail to meet expectations, the next most important factors are visual privacy, noise level, and colors and textures, respectively. According to the literature review of Kang et al. (19), office layout, air quality, thermal environment, lighting environment, and acoustic environment are five key IEQ aspects of open-plan offices. They all influence directly on workers' environmental satisfaction and work productivity.

According to our knowledge, workspace satisfaction has not previously been studied in relation to the social characteristics of the work community, such as atmosphere between colleagues or social capital. The most commonly used definition of social capital is that of Putnam (20), according to which social capital means trust, norms and networks that promote cooperation and improve the efficiency of society by facilitating its activities. Traditionally, social capital has been studied in residential or geographical areas, but scholars have also investigated social capital at work. In the work community, social capital includes support and encouragement, open communication and trust, and these things are also associated with well-being and economic efficiency. It also improves job satisfaction and reduces risk of burnout. It is also known that low self-perceived estimation of social capital as well as that perceived by colleagues is linked to the perceived deterioration of health, and consequently, to coping with work (21). According to Statistics Finland's (22) analysis, the better individual social capital is, the better is perceived health.

In this study, we aimed to determine employee satisfaction with a multi-space office. We also aimed to study the associations between satisfaction with working spaces and well-being. The hypothesis we consider is: Satisfaction with working space is associated with (1) self-satisfaction, (2) better recovery, (3) good perceived future work ability, (4) better working atmosphere, and (5) social capital.

## Methods

The office in this study was shared by six municipality-owned companies, who moved there in 2016, approximately a year before the research started. Thus, the research population was a newly formed work community, the members of which had previously worked in different kinds of offices ranging from open-plan offices to single-room offices. Some of the subjects had previous experience of open and flexible working environments, while others had not. The organizations did knowledge-oriented work and service work in the fields of regional development, regional planning, transportation, welfare services, infrastructure, trade, and innovation. The smallest organization had six employees, whereas the largest one had ~70. One of the organizations also had workers and contractors in other locations besides the shared office. The employees worked in different teams according to the varying demands of their work. Thus, we believe that good social capital might be an important factor affecting how satisfied employees are with a space solution that supports this kind of work.

The office space had recently been renovated and designed for the purposes of knowledge-oriented work. It was basically an activity-based office or a multi-space office. Some organizations practiced hot-desking, while others had fixed desks for each employee. The office space was divided by glass walls so that two of the organizations had separate areas, one of which was behind an inner door. Three of the organizations shared a large open-plan space. In addition to this, there were silent spaces, phone booths, and cubicles for small meetings, a large recreation area, and a conference center.

We emailed the questionnaire to all employees of the six organizations in the workspace in Webropol format. We sent it in January 2018, and a reminder twice in February. Information letter on voluntary participation, confidentiality, etc., was included in the survey. Every participant had the opportunity to ask anything about the survey in an information session. Participants knew that they could interrupt their participation at any time without cause. A written informed consent was not required as per local legislation and institutional requirements due to the assumption that by replying to the questionnaire they will give permission to analyzing the data. This study followed all guidelines and instructions of the Finnish National Board on Research Integrity and because we did not have individual health-related questions, ethical approval was not required according to local legislation and national requirements. This study was not medical research, and questions were not deeply related to individual health but rather to well-being.

The main outcome variable was a modified version of the Indoor Environment Quality questionnaire developed in the Center for the Built Environment at the University of California (7). We asked two questions about the amount and comfort of lighting. Two questions addressed acoustic quality and three questions addressed office layout. Two questions were about office furnishings, and one question elicited overall satisfaction. The respondents assessed their satisfaction level for each item on a seven-point scale ranging from “1 = very dissatisfied” through “4 = neutral” to “7 = very satisfied”. From the above-mentioned questions, we formed a sum variable describing general workplace satisfaction.

We selected six items to study social capital at work (23). These items were designed to assess social capital specifically in the work content. We excluded items about supervisors, because the respondents worked in six organizations and did not have the same supervisor. These items were: We have a “we are together” attitude, people keep each other informed about work-related issues in the work unit, people feel understood and accepted by each other, people in the work unit cooperate in order to help develop and apply new ideas, members of the work unit build on each other's ideas in order to achieve the best possible outcome. The response scales (1–5) ranged from: 1 = fully disagree; indicative of low social capital to 5 = fully agree, indicative of high social capital. The last item's scale was 1 = very little to 5 = very much.

The atmosphere in the office was elicited by a question from the Copenhagen Psychosocial Questionnaire (24): Is there a good atmosphere between you and your colleagues? The response options were on a five-point scale: 1 = never/very rarely… 5 = always/almost always. Self-satisfaction was elicited by the item: In general, I feel very positive about myself. The response options were on a five-point scale ranging from “strongly agree” to “strongly disagree” (25). Two questions were from the Finnish Work and Health interview study (26): (1) Do you believe that your health will allow you to work in your current occupation until retirement age: no, probably not, probably yes, yes, I cannot say. (2) Do you recover from work strain after your work day or work shift: well, moderately, poorly, I cannot say.

### Statistical Analysis

We used IBM SPSS 25-statistical software for the statistical analyses.

Before the analyses, we merged the categories of some variables (see below) because some categories had too few responses and we wanted to avoid the loss of statistical power.

The descriptive part of the results has been reported in terms of frequencies and percentages, averages, and minimum and maximum values.

We examined the relationship between workspace satisfaction and well-being using cross-tabulation and Pearson's Chi-Square. The statistically significant level was set to *p* < 0.05.

We created a sum variable from the questions describing workspace satisfaction so that the numerical codes for the 10 sub-questions were summed up and divided by the number of questions. Cronbach's alpha of the sum variable (describes the unity of questions) was 0.85. We also created sum variables for its different parts: lighting (α = 0.81), acoustics (α = 0.73), design (α = 0.61), and workspace furniture (α = 0.77). One question elicited general satisfaction with one's own workstation. The sum variable was also formed from the social capital variable's five sub-questions in the same way (α = 0.85).

Workspace satisfaction was described by a three-grade sum variable: dissatisfied (1–4), partly satisfied (4.1–5), and satisfied (5.1–7). The classification for a variable describing satisfaction with lighting, acoustics, design, and workspace furniture was dissatisfied (1–3), partly satisfied (3.1–5), and satisfied (5.1–7). Overall satisfaction with one's own workstation was categorized as: dissatisfied (1–2), partly satisfied (3–5) and satisfied (6–7). In cross-tabulations, we combined dissatisfied and partly satisfied into one class. The sum variable on social capital was: disagree (1–2.4), neither disagree nor agree (2.5–3), agree (3.1–5).

The classifications were based on the background information of the issue and mathematical calculations.

A model describing the variability of workspace satisfaction was created using linear regression analysis. In the model, the main outcome variable was the sum variable of workspace satisfaction. It was increased to the 2nd power, so that it had normal distribution. We standardized the model according to age and each person's own estimate of ability to work until retirement age. We wanted to have person's own estimate of future work ability as an individual characteristic in the model. Age was induced as it might affect the associations. We then added the well-being variables into the model alternately.

## Results

### Background Information

The response rate was 49% (*N* = 91). The respondents were from all the different sized organizations and their number varied by organization from 4 to 33. Most of the respondents were full-time and permanent workers. About one-fourth were temporary workers. Approximately, one-fifth of the employees (21%) had started working for the same employer in the fall of 2016 or later. The mean age of the respondents was 46 ± 11 years (between 26 and 62). Women comprised 68% (*n* = 62), men 26% (*n* = 24) of the respondents, and 6% (*n* = 5) identified as non-binary or did not answer the question. There were 16 supervisors (18%). More than half of the respondents (62%) reported having worked in the last 12 months outside the office. Only about one-fifth (22%) of the respondents worked at a fixed workstation, on a fixed computer and did not travel or telework at all.

### Satisfaction With Working Space

The respondents were most satisfied with the workspace furniture (82% of respondents) and lighting (71%) (Table 1). They were most dissatisfied with the acoustics of the workspace (44% of respondents). They were also satisfied with workspace design (54% of respondents) and their own workstation (53% of respondents). In general, according to the sum variable including all the sub-items of workplace satisfaction, more than half (57%) were satisfied with their workspace.

**Table 1:** Satisfaction with working space.

	**Dissatisfied** **% (*n*)**	**Partly satisfied** **% (*n*)**	**Satisfied** **% (*n*)**
Sum variable	16 (15)	26 (24)	57 (52)
Lighting	15 (14)	13 (12)	71 (65)
Acoustics	44 (40)	33 (30)	23 (21)
Workspace design	10 (9)	36 (33)	54 (49)
Workspace furniture	4 (4)	13 (12)	82 (75)
Satisfied with own personal workstation	14 (13)	33 (30)	53 (48)

Background variables were not related to workspace satisfaction, but the supervisors were more satisfied with their own personal workstation more often (81% of supervisors were satisfied with their own workstation and 49% of the other respondents respectively, *p* = 0.054).

### Associations Between Workspace Satisfaction and Well-Being

Background variables were also not associated with well-being variables, except perceived recovery. Men reported recovering well after the work day more often than women (67% of men recovered well and 41% of women, respectively, *p* < 0.05). Three-quarters of those in management or supervisor positions felt that they recovered well from work, compared to only 41% of those in other positions (*p* < 0.05).

Of those who were satisfied with their workspace, almost all were satisfied with themselves (94% of respondents) as well, whereas about three fourths (77%, *p* < 0.05) of those dissatisfied with their workspace were satisfied with themselves (Table 2).

**Table 2:** Associations between workspace satisfaction and well-being.

	**Satisfaction with working space**		
	**All**	**Dissatisfied or partly satisfied**	**Satisfied**
	**% (*n*)**	**% (*n*)**	**% (*n*)**
**In general, i feel very positive about myself** *p **<** **0.05**, ***χ***^**2**^ **=** **5.831**			
Disagree or neither disagree nor agree between	13 (12)	23 (9)	6 (3)
Agree	87 (79)	77 (30)	94 (49)
All	100 (91)	100 (39)	100 (52)
**My health will allow me to work in my current occupation until retirement age** **p **<** **0.01**, ***χ***^**2**^ **=** **8,794**			
Probably No/yes	38 (33)	57 (20)	26 (13)
Yes	62 (53)	43 (15)	74 (38)
All	100 (86)	100 (35)	100 (51)
**Recovery from work strain** **p **<** **0.01**, ***χ***^**2**^ **=** **10.944**			
Moderate/poor	53 (48)	74 (28)	38 (20)
Good	47 (42)	26 (10)	62 (32)
All	100 (90)	100 (38)	100 (52)
**Good atmosphere among colleagues in office** ***p **<** **0.001**, ***χ***^**2**^ **=** **20.992**			
Sometimes/rarely	30 (27)	54 (21)	12 (6)
Often	39 (35)	33 (13)	43 (22)
Always/almost always	31 (28)	13 (5)	45 (23)
All	100 (90)	100 (39)	100 (51)
**Good social capital** *p **<** **0.05**, ***χ***^**2**^ **=** **9.258**			
Disagree	37 (34)	54 (21)	25 (13)
Neither agree nor disagree	31 (28)	28 (11)	33 (17)
Agree	32 (29)	18 (7)	42 (22)
All	100 (91)	100 (39)	100 (52)

Self-perceived future work ability was more positive among those who were satisfied with their workspace than among the dissatisfied: Three out of four satisfied respondents believed they would continue until retirement age, whereas more than half (57%) of the dissatisfied were unsure (*p* < 0.01).

Those who were satisfied with the workspace felt that they recovered from their workload well (62% of satisfied), compared to 26% of those who were dissatisfied (*p* < 0.01).

Of those who were satisfied with the workspace, almost half (45%) also felt that they always or almost always had a good atmosphere between colleagues. In contrast, more than half (54%) of the dissatisfied and partly satisfied respondents sometimes or rarely experienced this (Table 2) (*p* < 0.001).

Those who were satisfied with the workspace more often had a high individual-level social capital than those who were dissatisfied (42% vs. 18% of respondents, *p* < 0.05).

### Satisfaction With Acoustics

The greatest dissatisfaction was with the acoustics of the workspace.

Most of those who were satisfied with acoustics (81%) believed they would be able to work in their profession until retirement age, whereas almost half of those who were dissatisfied (45%) were unsure (*p* < 0.05) (Table 3).

**Table 3:** Associations between satisfaction with acoustics and well-being.

	**Satisfaction with acoustic**		
	**All**	**Dissatisfied**	**Satisfied**
	**% (*n*)**	**% (*n*)**	**% (*n*)**
**In general, i feel very positive about myself** *p **<** **0.05**, ***χ***^**2**^ **=** **4.147**			
Disagree or neither disagree nor disagree between	13 (12)	17 (12)	0 (0)
Agree	87 (79)	83 (58)	100 (21)
All	100 (91)	100 (70)	100 (21)
**My health will allow me to work in my current occupation until retirement age** *p **<** **0.05**, ***χ***^**2**^ **=** **4.388**			
Probably no/yes	38 (33)	45 (29)	19 (4)
Yes	62 (53)	55 (36)	81 (17)
All	100 (86)	100 (65)	100 (21)
**Recovery from work strain** *p **<** **0.05**, ***χ***^**2**^ **=** **9.593**			
Moderate/poor	53 (48)	62 (43)	24 (5)
Good	47 (42)	38 (26)	76 (16)
All	100 (90)	100 (69)	100 (21)
**Good atmosphere among colleagues in office** **p **<** **0.01**, ***χ***^**2**^ **=** **11.480**			
Sometimes/rarely	30 (27)	39 (27)	0 (0)
Often	39 (35)	36 (25)	50 (10)
Always/almost always	31 (28)	26 (18)	50 (10)
All	100 (90)	100 (70)	100 (20)
**Good social capital** **p **<** **0.01**, ***χ***^**2**^ **=** **9.744**			
Disagree	37 (34)	46 (32)	9 (2)
Neither agree nor disagree	31 (28)	29 (20)	38 (8)
Agree	32 (29)	26 (18)	52 (11)
All	100 (91)	100 (70)	100 (21)

We found a strong association between dissatisfaction with acoustics and recovery from workload, clearly more than half (62%) of the dissatisfied respondents experienced poor or moderate recovery, whereas three quarters (76%) of those who were satisfied recovered well (*p* < 0.05).

More than half (52%) of those who were satisfied with acoustics had high individual-level social capital, compared to about a quarter of those who were dissatisfied (26%) (*p* < 0.01).

Those who were satisfied with acoustics also felt positive about themselves and enjoyed a good atmosphere. With these variables, the statistical test was not reliable because some categories had no answers.

### Model for Describing Satisfaction With Working Space

We tested which well-being variables best explain the variations in satisfaction with working space. We found that the social capital variable had the best explanation power as unstandardized and standardized by age and own estimate of future work ability (Table 4). The standardized model explained 24.8% of the variation.

**Table 4:** Models describing satisfaction with working space, unstandardized (step 1) and standardized (step 2).

	***n***	**B (95% CI)**	***p*-value**	**F**	**Adjusted R squared**
**Step 1 (Unstandardized)**					
Social capital	91	6.415 (3.93–8.90)	<0.001	26.338	0.220
Positive feeling about myself	91	9.156 (3.01–15.3)	<0.01	8.760	0.079
Future work ability	86	6.792 (2.55–11.04)	<0.01	10.117	0.097
Recovery	90	6.578 (2.40–10.75)	<0.01	9.809	0.090
Atmosphere	90	6.347 (3.87–8.82)	<0.001	25.934	0.219
Age	74	1.411 (−3.13–5.95)	NS	3.474	0.033
**Step 2 (Standardized)**					
**Model 1:**	72		<0.01	4.213	0.120
Future work ability		4.701 (0.23–9.12)	<0.05		
Age		0.175 (−0.03–0.38)	NS		
Positive feeling about myself		4.263 (−2.39–10.91)	NS		
**Model 2:**	71		<0.05	3.583	0.100
Future work ability		4.105 (−0.80–9.01)	NS		
Age		0.170 (−0.04–0.38)	NS		
Recovery		2.403 (−2.44–7.25)	NS		
**Model 3:**	71		<0.001	7.114	0.208
Future work ability		3.192 (−1.23–7.62)	NS		
Age		0.131 (−0.06–0.33)	NS		
Atmosphere		4.524 (1.56–7.48)	*p* < 0.01		
**Model 4:**	72		<0.001	8.796	0.248
Future work ability		4.477 (0.40–8.56)	<0.05		
Age		0.123 (−0.066–3.11)	NS		
Social capital		4.772 (2.18-7.36)	<0.001		

## Discussion and Conclusions

### Summary of Results

The employees were most satisfied with the workspace furniture (82% of respondents) and the most dissatisfied with workspace acoustics (44% of respondents). Workspace satisfaction was associated with good self-satisfaction, good perceived future work ability, and recovery. Of the social factors of workspace satisfaction, we found associations with good atmosphere among colleagues in the whole office and good social capital. Satisfaction with acoustics was also associated with good self-perceived future work ability and recovery, in particular with high individual-level social capital. Social capital best explained the general variation in workplace satisfaction.

### Satisfaction With Working Space

In line with the results of Kim and Dear (7) concerning the same kind of office environment, the respondents in this study were most dissatisfied with the acoustics of the workspace, especially with the lack of audible privacy. In both studies, the respondents were most satisfied with the ease of interaction with colleagues. A completely accurate comparison between these two studies is not possible due to their different analytical and reporting methods.

### Satisfaction With Working Space and Its Association With Well-Being (at Work)

Workspace satisfaction was associated with recovery from workload. Perhaps when one is satisfied with one's workspace and its functionality, this is also reflected in good recovery after the work day. Good recovery was also associated with acoustic satisfaction. Not many studies have examined recovery while working in different kind of workspaces. However, in a study by Lindberg et al. (14), both the perceived and measured indicators showed that those working in open offices are more physically active and less stressed. This may be one explanation for the good recovery found in this study; when moving from one place to another is well planned and appropriate, it increases satisfaction and promotes recovery. Therefore, attention should be paid to planning workspaces so that they promote recovery. This is also highlighted by the results of Niip et al. (27), who found that perceived fatigue increased after moving to work in a multi-space office. In addition, self-satisfaction, and perceived ability to cope with work in the future were associated with workspace satisfaction. This suggests that the comfort and functionality of workspaces can contribute to individual reflection on coping and continuation at work. Further research is needed to confirm this association.

We found a strong association between workspace satisfaction and a good atmosphere in the workplace community, not just between colleagues in their own organization. The good side of working in multi-space offices is the increased possibility of interaction with colleagues, so the importance of an atmosphere that supports good interaction is essential. However, Bernstein and Turban (11) found that measured face-to-face interaction decreased after moving to work in more open workspaces, and that at the same time, electronic interaction increased. Could it be that even if interaction decreases, a good atmosphere still plays an important role in satisfaction? People may trust each other and not have to think about, for example, whether someone is monitoring their work, who talks to who, and so on.

Self-perceived future work ability and recovery were associated with acoustics satisfaction. The association between social capital and acoustics satisfaction was even stronger than the association between social capital and general workplace satisfaction. How can an acoustic work environment be improved, and thus also well-being? The attenuation of disturbing noises has been studied and technical solutions recommended [e.g., (28, 29)]. Could social interaction increase social capital and thus increase satisfaction with the working space in terms of tolerance of its acoustic properties? It may be that people with good social interaction are interested in other people and it is natural for them to work in a noisy multi-space office whereas for some people this is not at all natural. This is in line with the research by Gerdenitch et al. (16) of perceived need-supply fit in activity-based office. In any case, joint development of working practices improves the comfort of working in shared working spaces. De Been et al. (10) conclude that the behavioral issues of both managers and employees in solving problems are important to consider in the form of common rules, for example. Di Blasio et al. (8) reports that the majority of employees (70%) are willing to adjust their voice levels, using, for example, feedback from noise monitors. Work-related development measures should also be managed systematically and collectively.

### Social Capital and Its Association With Satisfaction With Working Space

The results of this study support our hypothesis of the association of social capital with workspace satisfaction. However, based on this study material, we cannot say whether good social capital increases workspace satisfaction in a multi-space office solution or whether workspace satisfaction increases social capital. However, these things are associated with each other. Thus, social capital can be supported by designing (joint) functional workspaces appropriately.

Research is needed on whether a change in work in the form of, for example, a new working space solution can act as a source of inspiration and increase social capital. Associations between multi-space offices and well-being are interesting, not only in terms of job satisfaction, but also in terms of recovery and better perceived work ability, and these things should be identified as the basis for planning.

### Limitations and Strengths of Study

We did not ask about the content of work thoroughly enough. The content of work may have varied somewhat, both within and between organizations, which may have affected the responses. However, all the participants did expert/knowledge work. It may be more difficult to do planning tasks that require concentration in a multi-space office, whereas interactions and coordination are emphasized in customer service work, to which a multi-space office might be better suited (2). Only about one fifth of the respondents reported constantly working at a fixed workstation; others traveled or sometimes worked out of office. Almost all (about 80%) had worked at the workplace since its establishment, so the working period most likely did not have a large impact on the answers.

Only about half of the employees (49%) of all the six organizations responded to the questionnaire. However, the respondents were from all the organizations and the age range was rather large, so the results can be considered at least preliminary. The modeling of workspace satisfaction was hampered by the fact that many respondents did not report their age, and that the number of supervisors and workers per organization was small.

The analysis used in this study does not reveal the direction of the effects. It might be that when a workspace is considered good, well-being improves; or that those who have good well-being are more satisfied working in a multi-space office environment.

## Conclusions

Workspace satisfaction, and individual as well social well-being, measured in many different ways, and especially social capital, are associated with each other. It is worth paying attention to the design, functionality, and management of the used workspaces, in order to maintain workplace satisfaction and well-being.

## Data Availability Statement

The datasets generated for this study will not be made publicly available for ethical reasons. We do not have the permission of the organizations involved in the research.

## Ethics Statement

Ethical review and approval was not required for the study on human participants in accordance with the local legislation and national requirements. Written informed consent for participation was not required for this study in accordance with the national legislation and requirements.

## Author Contributions

SL, SK, HA, and PH contributed to the study conception and design, data collection and interpretation, manuscript preparation and review. JU contributed to analyzing and writing the results.

### Conflict of Interest

The authors declare that the research was conducted in the absence of any commercial or financial relationships that could be construed as a potential conflict of interest.
